# Inequities in Forgone Medical Care and Health Insurance in a Key Geopolitical Area Along the US–Mexico Border

**DOI:** 10.3390/healthcare13182295

**Published:** 2025-09-13

**Authors:** Samuel D. C. Towne, Wei Li, Chanam Lee, Minjie Xu, Jiahe Bian, Leah D. Whigham, Marcia G. Ory

**Affiliations:** 1 School of Global Health Management and Informatics, University of Central Florida, Orlando, FL 32816, USA; 2 Disability, Aging, and Technology Cluster, University of Central Florida, Orlando, FL 32816, USA; 3 Department of Environmental and Occupational Health, School of Public Health, Texas A&M University, College Station, TX 77843, USA; mory@tamu.edu; 4 Southwest Rural Health Research Center, Texas A&M University, College Station, TX 77843, USA; 5 Center for Community Health and Aging, Texas A&M University, College Station, TX 77843, USA; 6 Landscape Architecture and Urban Planning, Texas A&M University, College Station, TX 77843, USA; wli@tamu.edu (W.L.); chanam@tamu.edu (C.L.); 7 Air Quality and Environment Division, Texas A&M Transportation Institute, Austin, TX 78752, USA; mxu@tamu.edu; 8 College of Environment and Design, University of Georgia, Athens, GA 30602, USA; jbian@uga.edu; 9 Department of Nutritional Sciences, The University of Texas at Austin, Austin, TX 78712, USA; leah.whigham@austin.utexas.edu

**Keywords:** social and structural determinants of health inequities, public health, health inequities, US–Mexico border, disease risk factors, geopolitical boundaries

## Abstract

Background: Residents of the US–Mexico border face cost-related barriers in accessing necessary medical care. Given the potential for individualized or broader tailoring of solutions to reflect community needs, we sought to identify risk factors for being uninsured and forgoing necessary medical care due to cost among a largely Hispanic adult population residing along the US–Mexico border. Methods: Surveys among adults in a major US–Mexico border area were used to investigate cost-related forgone medical care and lack of insurance. Binary Logit models were employed to model multiple binary outcomes informed by our theoretical frameworks. Results: Lower education, Hispanic ethnicity, being younger, lacking underlying illness and/or having obesity, forgoing medical care due to cost, and having lower income were associated with a higher likelihood of being uninsured; while being female, being younger, having underlying illness and/or having obesity (potential increased risk of severe illness due to COVID-19), lacking insurance, and having a lower income were risk factors for forgone medical care due to cost. Conclusions: This study adds novel insight into existing health inequities facing those residing along the US–Mexico border region, thereby holding timely public health implications.

## 1. Introduction

Equitable access to health care is the goal of multiple key partners, yet broad social and structural determinants of health inequities face millions globally [1]. Thereby, elucidating factors associated with equitable access to critical medical care, including forgone medical care, that have the potential to be tantamount in many ways to delivering on the goals of public health [2], is a major priority. In particular, the cost of health care, among many reasons, is a major barrier to accessing necessary health care. Past work identified a higher likelihood of forgone medical care among adult individuals from a racial or ethnic minority group [3], individuals who were not insured [3], individuals with lower education [3], and with low English language proficiency [4]. Cost-related forgone medical care, the major focus of the current study, is among the major factors affecting the lives of many in the US [3,4], where the health care system can present costly barriers to accessing necessary health care, particularly among at-risk populations.

In the absence of barrier-free access to necessary medical care, continued surveillance of forgone medical care is necessary, especially for potentially higher-risk areas of the US, including border areas. Border areas between nations can be host to health disparities, as is the case along the US–Mexico border [5,6,7,8,9], where early research from nearly 20 years ago (in El Paso County) identified having health insurance as being significantly associated with access to health care among a largely Hispanic population [10]. More recent evidence, also provided evidence of gaps in access to health care along US–Mexico border communities for Hispanic adults [11].

Furthermore, in terms of need-related factors associated with access to health care more generally, Hispanic residents had higher rates of certain chronic diseases (e.g., diabetes) as compared to White residents in nationwide analyses [12]. Therefore, identifying progress or lack thereof in equitable access to health care is both complex and of critical importance to continued public health surveillance efforts in major US–Mexico border areas, where large proportions of Hispanic individuals reside.

The previously identified existing inequities in access to health care, coupled with high rates of disease (e.g., diabetes), are also timely in terms of the past global COVID-19 pandemic and the ongoing endemic [13], which impacted this region significantly. Considering the study timeline, we also identify populations that may be at higher risk of severe illness due to COVID-19 [14], given El Paso County (Texas) experienced a significant rise in COVID-19 cases in late 2020 with over 90,000 positive cases with a cumulative positivity rate of over 13% as of 8 December, 2020 [15] and as of mid-March 2021 over 126,000 positive cases were reported with over 2200 deaths [15]. In terms of the pandemic, chronic disease was prevalent among underlying conditions in those hospitalized for COVID-19 [16]. Other recent evidence suggests that, when assessing COVID-19-associated hospitalizations, individuals of minority race or ethnicity, as compared to non-Hispanic White individuals, had higher hospitalization rates [17]. Furthermore, other analyses using national survey data to characterize risk of severe illness due to COVID-19 indicated disparities across race, particularly facing individuals identifying as Black or African American, as well as American Indian, compared to individuals identifying as White [18]. Furthermore, a cross-sectional study, using national survey data from prior to the pandemic to assess potential risk among health care workers in the US during the pandemic, used self-reported measures (e.g., age, chronic medical conditions) to estimate potential risk for severe illness if contracting COVID-19, finding millions of health care workers were potentially at risk [19]. This prior study supports the critical need for ongoing public health surveillance as it allowed for an estimate of risk among a designated population, using self-reported risk factors as a way to, among other things, plan for allocating resources in a given population or area. The study further characterized additional burden related to being unable to afford medications and being uninsured, which may further complicate accessing necessary medical care [19]. Another study sought to estimate forgone medical care during the pandemic, using a national online survey of adults in the US, finding nearly 41% reported avoiding or delaying medical care because of concerns related to COVID-19 [20]. This past study further identified groups that may be at higher risk of avoiding urgent or emergency care, finding that persons with disabilities, young adults, Hispanic adults, Black adults, unpaid caregivers, and individuals with underlying medical conditions had a higher risk [20]. The authors concluded that, among other things, if this avoidance behavior continues, it may have lasting impacts on, for example, chronic disease self-management, early detection of new medical conditions, etc., and that having this knowledge can inform future strategic medical care delivery and outreach or communication efforts [20]. The potential coexistence of previously identified health inequities along the US–Mexico border [8,9,21] and more recent examples of potential health inequities across race and ethnicity from COVID-19-related data [17] more broadly, highlight a need for continued public health surveillance among potentially at-risk populations, even absent a pandemic.

### 1.1. Aims

Given the past work highlighting gaps in access to care for potentially vulnerable populations [1], especially along the US–Mexico border, we sought to examine factors associated with health inequities in one major border city. We primarily sought to answer the research question of how (if at all) social and structural determinants of health inequities influence both forgone medical care and whether or not one has health insurance. This line of inquiry was supported by multiple theoretical frameworks, including the WHO Framework for Action on the Social Determinants of Health [22] and the NIA Health Disparities Research Framework [23] and the NIMHD Research Framework [24]. Moreover, the current study also incorporates a major international boundary, with potential cross-country medical access, a population that is primarily Hispanic, and takes place in a state that has failed to adopt Medicaid Expansion (via the ACA, 2010), and also at a time when research dollars for work on health inequities are increasingly uncertain, making this study relevant to multiple timelines (e.g., pre-COVID-19, present day) and individuals with an interest in improving population health.

### 1.2. Expected Contribution of the Current Study

This study adds updated evidence of health inequities while adding insights into relevant risk factors, such as underlying illness and/or obesity, that can characterize at-risk populations in a unique geospatial location with a history of health inequities [5,6,7,8,9]. Public health surveillance efforts along the US–Mexico border remain a critical need, given existing inequities, but also due to the ongoing complexity of seeking necessary medical care along a major international border crossing. Therefore, this study will add to the existing scientific knowledge about gaps in access to care, while also providing data for those seeking to better characterize and reduce the burden of health inequities among potentially at-risk populations along the US–Mexico border.

## 2. Methods

This study utilized survey data (online = 2240; paper = 845 participants) collected from El Paso, Texas, in 2018–2019 as part of an NIH-funded study focused on Hispanic adults residing in this border area. Both English language and Spanish language surveys were utilized, depending on the preference of the participant, with written informed consent. Inclusion criteria included age (20 and older) and residence (within El Paso, Texas). As of 2019, the population of El Paso was at 681,728, with 80.9% Hispanic, 79.6% with high school or higher education among those aged 25 years and older, and 21.6% without health insurance among those under 65 years, with a median household income of USD45,656 [25]. In terms of sampling, data were gathered from a rigorous design that incorporated geographic stratification to increase comparability to the local community and address selection bias [26]. For example, the percentage of individuals reporting Hispanic ethnicity, at ~84% overall (see Table 1), was similar to that of El Paso at ~81% overall. Also, given the unique aspects that may come with recruiting from a community with a predominantly Hispanic demographic, we considered several community-engaged and culturally sensitive recruitment strategies (e.g., extensive pilot testing among the research team and community liaisons and utilizing ongoing community partnerships in the local area).

### 2.1. Dependent Variables

Forgone medical care due to cost was operationally defined as responding ‘yes’ to the question “Was there a time in the past 12 months when you needed to see a doctor but did not because of cost”, which was modeled off the Behavioral Risk Factor Surveillance System (BRFSS) [27] and referenced in the PhenX Toolkit [28]. In addition, we assessed insurance status (uninsured versus insured, including private health insurance, Medicaid, Medicare, Military health insurance, and other) as both a dependent variable and independent variable (when modeling forgone medical care). This measure of forgone care was based on a single survey item, which has been used by the longest running survey in the world, for many years [29], with comparisons to other national surveys indicating variation due to, in part, data collection methods [30] and variation in recruitment methods (e.g., use of cell phones and landlines in the BRFSS as compared to face-to-face interviews in other surveys) [31] and utilized in multiple national studies [32,33,34].

### 2.2. Sociodemographic Variables

Multiple variables were included related to the WHO Framework for Action on the Social Determinants of Health [1] that are used to assess health inequities. These and other variables, served as measures of key social determinants of health inequities and were introduced in the models based on this and complementary theoretical frameworks. Variables included English language proficiency or ELP (high, low); education (less than college degree; college degree); ethnicity (Non-Hispanic; Hispanic); sex (female; male); age group (20–44 years; 45–64 years); income (less than USD60,000; at/above USD60,000) split roughly at the median household income for the United States which was USD60,293 (in 2018 dollars), 2014–2018 as reported in the US Census Quick Facts [35]; and insurance (uninsured; insured).

Furthermore, given the study setting, namely the burden of COVID-19 affecting residents of El Paso, several existing measures were utilized to construct a measure of health status (e.g., illness and/or potential risk of severe illness). In addition to overall health status, this variable may also shed insight into risk factors for severe illness if contracting COVID-19 [14], while *not* meant to perfectly reflect any/all relevant risk factors for a higher likelihood of having severe illness, *assuming* one were to contract COVID-19. Body Mass Index (BMI) at/above 30 (obesity) calculated using self-reported height and weight [14], and existing illness, including diabetes, cancer, asthma (of note, we captured asthma without knowing the severity of asthma, where moderate-to-severe is noted as a potential risk factor for severe illness due to COVID-19) [14], cardiovascular disease, being immunocompromised, chronic kidney disease being treated with dialysis, liver disease, heart failure, and high blood pressure, were included to characterize the possible risk of severe illness if contracting COVID-19 [14] and serve to characterize overall health status, referred to as having an underlying illness, including obesity.

### 2.3. Statistical Analyses

SAS 9.4 with SAS Studio (v15.1) was used for all statistical analyses. Descriptive statistics were presented to identify the distribution of the responses. Binary logistic regression was used with odds ratios and corresponding 95% Confidence Intervals to determine statistical significance. The choice of the analytical model was based on published statistical and methodological considerations, such as the distribution of the dependent variable, as a binary variable [36,37] (original form, i.e., not dichotomized from a more detailed measure), and that logistic regression has been described as the standard method for analyzing binary dependent variables across fields [38].

Both unadjusted and adjusted (multivariable) analyses were presented. In multivariable analyses, driven by our theoretical framework [1], multiple variables were simultaneously added to the model, including education, ethnicity, sex, age group, income, insurance status (when modeling forgone care due to cost), forgone care due to cost (when modeling insurance), and underlying illness and/or obesity.

We further tested for multiple interactions within the forgone care model, including the interaction of ethnicity and underlying illness and/or obesity; the interaction of ethnicity and education; the interaction of ethnicity and age; and the interaction of ethnicity and sex; all of which were not significant using a threshold of <0.05. These interactions were tested, given the unique setting and distribution of Hispanic individuals in the sample, yet we found no evidence of differential outcomes, and present analyses without the additional interactions [39]. We further explored potential variation in insurance type (no insurance versus: Private; Medicare (reduced sample due to age exclusions); Medicaid; Military Insurance; and other insurance. When including comparisons from each type of insurance to no insurance, we identified a similar trend. While briefly reported in the text, we present the simplified binary variable in adjusted analyses [40], given the similar patterns identified.

Models with income were presented separately, given the limited distribution of the data, the major outcome is cost-related, and due to the level of missingness for income. Furthermore, given the distribution of the data and that insurance status and forgone medical care were the primary outcomes, we excluded analyses that included those aged 65 and older (roughly 12% of the larger sample), who likely qualify for Medicare [41]. Thus, the focus on the primary analyses is on working-aged individuals (ranging from 20 to 64 in the current analyses). Our analytical sample of working-age adults was made up of 2152 respondents without missing data for major variables included in primary analyses. Additionally, ELP and car ownership were only included in descriptive and bivariate analyses given the limited distribution of those variables (see Table 1). Given we did not have evidence to suggest missing data were systematic across multiple variables, observations with missing data were removed, with complete case analyses performed [42].

## 3. Results

### 3.1. Paper Versus Online Survey—In Brief

When initially comparing missingness across multiple key variables, we identified that the paper survey had higher rates of missingness than the online version, yet the overall percentage was low for several key variables at less than ~2% for age and sex, and less than ~4% for education. However, there were higher rates of missing data across ethnicity, with the paper version having just over ~12% as compared to less than ~1% for the online version.

### 3.2. Descriptive Statistics

Among those aged 20–64, nearly 9-in-10 participants reported a high English language proficiency, 60% did not have a college degree, 84% reported Hispanic ethnicity, 64% were female, 60% were between the ages of 20–44 years, 82% had household incomes less than USD60,000, and 60% had underlying illness and/or obesity that may indicate a higher risk of severe illness if contracting COVID-19 based on available information as reported by the US CDC at the time of these analyses [14]. Furthermore, 38% were uninsured and 37% reported forgone medical care due to cost at least once in the past 12 months.

In addition to identifying the distribution of our two major outcomes of interest by underlying illness and/or obesity status, we also conducted additional distribution analysis by ethnicity, given that it was a major indicator of interest and that approximately 84% of respondents reported being Hispanic. When stratifying ethnicity by underlying illness and/or obesity, we found roughly 60% of those who were non-Hispanic and 60% of those who were Hispanic had underlying illness and/or obesity (see Table 1 notes).

*Unadjusted*

*Uninsured*. Multiple factors were associated with a higher likelihood of being *uninsured*, including lower English language proficiency (versus high); not having a college degree versus higher educational attainment; being Hispanic (versus non-Hispanic); being aged 20–44 (versus 45–64 years); *not* having a BMI of 30 or higher and/or underlying illness; forgoing medical care due to cost (versus not); not having a car, and having a household income of less than USD60,000.

*Forgone care*. In our initial examination, we identified that when any insurance type was compared to no insurance, there was a statistically significant lower likelihood in the odds (all ORs <1.0 with 95% Confidence Intervals) that one reported forgone medical care, indicating that any insurance type tested was protective against forgone medical care due to cost when compared to the absence of insurance. As noted in the Methods Section, we present the dichotomized measure in the unadjusted and adjusted analyses (as presented in Table 1, Table 2 and Table 3).

Factors associated with a higher likelihood of *forgone medical care due to cost in the past year*, included not having a college degree versus higher educational attainment; being Hispanic (versus non-Hispanic); being female (versus male); being aged 20–44 (versus 45–64 years); having a BMI of 30 or higher and/or underlying illness; lacking insurance; and having a household income of less than USD60,000.

*Adjusted*

*Uninsured*. We identified lower education, Hispanic ethnicity, being younger, *not* having a BMI of 30 or higher, and/or underlying illness, and forgoing medical care due to cost as being associated with a higher likelihood of being uninsured. Similar findings were identified for the model that additionally included income, where lower income was also associated with a higher likelihood of being insured. When additionally testing for an interaction of ethnicity and underlying illness and/or obesity, we found no evidence of a differential outcome (*p* > 0.05).

*Forgone care*. Upon examining for multiple interactions within the forgone care model, we found no evidence of statistically significant interactions. These included the interaction of ethnicity and underlying illness and/or obesity (*p* > 0.05); the interaction of ethnicity and education (*p* > 0.05); the interaction of ethnicity and age (*p* > 0.05); and the interaction of ethnicity and sex (*p* > 0.05). After removing interactions (due to no evidence of a significant relationship), we present the model without interactions.

We identified being female, being younger; having a BMI of 30 or higher, and/or underlying illness as operationally defined in the current study; and lacking insurance as being associated with a higher likelihood of forgoing medical care due to cost. Similar findings were identified for the model that additionally included income (smaller sample size with income included due to missing data—see Table notes), where lower income was also associated with a higher likelihood of forgoing medical care due to cost.

## 4. Discussion

The current analysis adds to the existing literature about risk factors for lacking insurance and also forgoing medical care due to cost in the past year, among a particularly unique geographic area within the US. The US–Mexico border is unique in many ways [8,9], which warrants analyses with a focused geographic scope than might otherwise be presented in larger national or even state-wide analyses, thereby adding significant value to the current analyses. Our analyses also build upon past work highlighting disparities in risk factors for severe illness due to COVID-19, which has recently been published, albeit using data from 2011, where high rates of risk factors for severe COVID-19-related illness were identified [43] in El Paso, which complements the findings in the current study. Our study was able to utilize more recent data collected nearly ten years later and provide complex multivariable analyses that takes multiple variables into consideration that have been highlighted in the WHO theoretical framework [1] related to social and structural determinants of health inequities and also complementary frameworks [23,24]. Furthermore, forgone medical care has also been investigated within the pandemic timeline. Somewhat recent experimental data from the U.S. Census Bureau Household Pulse Survey (Week 27) suggests that Hispanic individuals, both nationally and in Texas, respectively, had a higher rate (29% and 30%) of delayed (forgone) medical care *due to the pandemic within the past month* than those who reported being non-Hispanic White (23% and 22%) [44]. This is compared to roughly 38% of Hispanic individuals and 32% of White individuals reporting forgone medical care *due to cost in the past year* in the current study. While assessing somewhat different outcomes across different timelines and different areas, the identified higher rates among Hispanic individuals were relatively consistent.

Furthermore, complementing the major analyses is the additional inclusion of a measure of underlying illness and/or obesity, which may suggest a potentially higher risk for severe illness due to COVID-19 [14], especially among those who may be unvaccinated. While this measure is not without limitations given new knowledge of risk factors for severe illness associated with COVID-19 may emerge, it does independently provide insight into risk factors of lacking insurance and forgone care among those with underlying illness and/or obesity regardless of potential insight into COVID-19 risks.

Our findings are also relevant in light of previous work [4,10,11,45,46,47,48,49,50,51,52,53,54,55,56,57,58,59,60,61,62,63,64,65,66], though a full review of the existing literature is outside the scope of this study. For example, while limited to bivariate analyses in the current study due to being highly skewed, limited English language proficiency has been shown to be associated with forgone medical care using national survey data from the US [4]. Similarly, in the current study, low English language proficiency was associated with being uninsured in unadjusted analyses, but not with forgone medical care due to cost in the past 12 months. Our finding of the relationship between lacking health insurance and a higher likelihood of forgone medical care due to cost is also generally consistent with the early research in El Paso County that identified having health insurance as being associated with access to health care [10]. Further, past research cited a strong link between lower affordability of health care and the absence of health insurance among Hispanic individuals [51]. Additionally, a large multi-year nationally representative study identified some potential positive benefits of access to care among individuals with lower income, specifically Medicaid coverage, as being associated with a higher awareness and control of hypertension and being aware one was overweight [47]. While some have suggested the link between access to care, namely health insurance, and health may be nuanced and in need of further study [48], it is nonetheless an important indicator to consider when assessing health inequities.

Policies that seek to provide access to health insurance for more vulnerable adults with lower income, such as Medicaid Expansion under the Affordable Care Act (ACA) of 2010, may hold relevance. However, to date, Texas has not rolled out Medicaid Expansion across the state [67]. If implemented, according to a recent study, it may allow for an estimated 1.2 million uninsured persons to be eligible [68]. Moreover, a systematic review (published in 2018) of the effects of Medicaid expansion associated with the ACA identified that Medicaid expansion was linked to increased health insurance coverage, care quality, and service utilization, among other items [69]. Another evaluation of Medicaid Expansion identified additional benefits associated with expansion, including, but not limited to, improved health outcomes among individuals identified as having low-income and also reductions in mortality [70]. Thus, there is evidence to suggest positive population-level benefits associated with this policy implementation. Policy makers may use this study and others [67,68] when considering the implementation of Medicaid Expansion in Texas.

In addition, while not the focus of the current study, previous research has suggested some potential for cross-border utilization of health care [71], namely in Mexico by Hispanic adults residing in the US, that may be related, in part, to low English language proficiency and/or an absence of consistent health insurance coverage [55]. Our measure of forgone medical care due to cost was *not* limited to care that would have *only* been sought within the US, and as such, may account for forgone care due to cost regardless of the location. Policies that seek to reduce the potential gaps in access to care, including, but not limited to policies addressing gaps in health care coverage [72], are needed to reduce health inequities facing potentially vulnerable populations along the US–Mexico border.

Further, other prior work has examined inequities across different population groups, with increased inequities in forgone medical care facing individuals reported as African American as compared to individuals reported as White along the Great Recession timeline [73]. Other work also indicates potential variation in health care utilization across socioeconomic status, with lower use among individuals with lower socioeconomic status outside the US [74]. A study from the US using the BRFSS to assess forgone medical care due to cost also reinforced this, with evidence that personal incomes were related to disparities in forgone care [32]. That study further reported that additional efforts may be needed to address disparities in forgone medical care due to cost, especially among individuals reported as Hispanic, given that they found high rates of forgone medical care among individuals reported as Hispanic, despite being more likely to be in areas described as having “more generous access policies”, as compared to other groups [32]. The authors concluded that among these considerations, that maintaining safety-net use and funding may be critical to increasing access to medical care [32]. Finally, the importance of increased focus on populations that may be more vulnerable to negative outcomes associated with COVID-19 mirrors, in some ways, the broader topic of social and structural determinants of health inequities where individuals with lower socioeconomic status may be more at risk for a variety of factors [75,76].

### Limitations

This cross-sectional analysis prevented assessing causality. This sample, while large enough for detailed sub-group analyses among multiple key characteristics, may not be representative of all US border areas along the US–Mexico border, as participants were sampled from specific geographically targeted areas. That said, the percentage of Hispanic individuals in our sample and that estimated in the US Census data in El Paso were similar, and so too was the percentage of those aged 65 and older. Thus, this sample may be somewhat representative of the city of El Paso. The inclusion of risk factors for severe illness due to COVID-19, as reported by the US CDC at the time of analyses [14] (which may change over time with new data, etc.), was limited to self-reported underlying illness and having a BMI classified as obesity calculated through self-reported height and weight. This measure was not purposefully created to perfectly assess all risk factors that may make individuals at higher risk of severe illness due to COVID-19 and as such is not a fully inclusive categorization of risk of severe illness if one were to contract COVID-19. Furthermore, data were collected prior to vaccine availability for COVID-19, and as such, risk characterization specific to COVID-19 may be most relevant among unvaccinated individuals. Further, the measure used to assess forgone medicl care did not allow for a measure of the intensity of forgone medical care, but only whether it occurred at all. In addition, this measure did not account for whether one actually sought care at all in the past 12 months and was reliant on recall. Furthermore, recall bias is a concern in survey research as one is likely forced to rely on one’s own memory to recall specific instances that one may be asked. However, this particular survey item (forgone medical care due to cost) asks about whether one experienced forgone medical care at least once in a defined timeline of 12 months and, as such, may be less prone to recall failures than might be captured in more complex survey items. Furthermore, it is also possible that individuals may over- or under report certain health-related behaviors due to social desirability [31]. However, there is also some work indicating that, when compared to in-person interviews, individuals taking a survey via computer-assisted self-administered interview may be more prone to share sensitive information, making the self-administered survey potentially less prone to social desirability bias [77]. Furthermore, given that we collected data via paper and online, we should note that some prior work noted limited or no variation in response rates, but some additional missing data among those responding via paper surveys [78]. This was consistent with the findings in the current study, in that higher rates of missing values were identified in the paper survey. That said, this measure was modeled off the Behavioral Risk Factor Surveillance System (BRFSS) [27] as well as being used to identify forgone medical care due to cost in past research [3], which is a major strength of the current study. Finally, complete case analyses is not without limitations, with multiple imputation as another common approach, yet with missing at random, both approaches may have minimal bias due to missing data [42]. These and other limitations are important to consider when interpreting the implications of this study.

## 5. Conclusions

This study identified health inequities along a major geopolitical boundary (the US–Mexico border), adding insight towards international health issues affecting a major economic area important for residents of the US and Mexico. Respondents who are more likely to lack insurance were with lower education, Hispanic, aged 20–44 versus roughly middle-aged (45–64 years), without underlying illness and/or obesity, reporting forgone medical care due to cost in the past year, and with lower incomes. Individuals more likely to have forgone medical care due to cost were female, aged 20–44 versus 45–64 years, uninsured, having underlying illness and/or obesity, and with lower incomes.

This study provides timely insight by identifying health inequities in two major outcomes related to access and utilization of health care, namely, lacking health insurance and also forgoing medical care due to cost and related risk factors. Our finding that those with underlying illness and/or obesity were more likely to forgo care creates a sense of a “double jeopardy” scenario that materialized during the pandemic. This evidence reinforces the utility of continued surveillance into risk factors for lacking insurance and forgoing medical care within a geopolitical boundary area with a history of persistent health inequities.

An influx of necessary and personalized resources to the area, considering unique community needs, is needed. This can include support for public policies targeted at an increase in the share of insured (e.g., via state Medicaid Expansion) and other less traditional health-related public polices (e.g., transportation policies related to increasing access to affordable, attractive, and efficient public transportation to reach health care resources), as well as community outreach campaigns to increase awareness of—and enrollment in—affordable options for health care.

Based on these empirical findings, examples of potential interventions may include, but would not be limited to, public awareness campaigns to increase awareness of affordable options for insurance, especially during any open enrollment period for state health insurance exchanges (marketplace) with the addition of trusted community organizations (that could help lead these campaigns and/or provide locations for these events, where appropriate). This may also include extending public insurance coverage (e.g., Medicaid expansion, dependent coverage) to reduce the prevalence of underinsurance and uninsurance, especially to previously ineligible individuals who have risk factors for forgone medical care and chronic disease and other relevant risk factors. Additionally, this effort may include tailored and culturally sensitive marketing material that is tailored to local needs (e.g., printed in multiple languages, including culturally sensitive material). Furthermore, clinical outreach by trusted health professionals in the local area with seminars on the importance of regular checkups for preventative care could be another component. To complement this, expansion of evidence-based community wellness programs that focus on chronic disease self-management for individuals with existing chronic conditions may provide additional community benefit. Methodologically, including geographic information systems (GISs) when assessing need and current allocation of resources by geography may be a critical resource with rich contextual data that can assist in asset allocation efforts, especially given finite resources across multiple sectors. Expanding community-based partnerships to be inclusive of particularly at-risk groups, including partnerships with trusted organizations that can provide culturally sensitive engagement, which may also include increased funding for individuals who can assist in navigating available resources (e.g., translators, individuals familiar with the region and specific context-based awareness of the US–Mexico border). These resources can be tailored to an individual’s needs but also personalized to be able fit into varying and unique *personalized community needs*, which will likely add significant value. Clearly, continued efforts are needed to re-assess progress, including benchmarks in key areas including rates of forgone medical care and insurance saturation.

## Figures and Tables

**Table 1:** Distribution of sample by key variables related to insurance status and forgone medical care due to cost.

		Aged 20–64 Years				
		Uninsured		Forgone Medical Care Due to Cost		Total
		No	Yes	No	Yes	n = 2126
English language proficiency	High	1237	629	1177	689	1866
	Low	79	175	168	86	254
Education	Less than College	679	598	763	514	1277
	College Degree	640	209	584	265	849
Ethnicity	Non-Hispanic	271	70	233	108	341
	Hispanic	1048	737	1114	671	1785
Sex	Female	834	516	817	533	1350
	Male	485	291	530	246	776
Age Group	20–44 years	742	541	764	519	1283
	45–64 years	577	266	583	260	843
Income	<USD60 K	904	675	917	662	1579
	≥USD60 K	316	38	293	61	354
Car Ownership	No Car	113	128	139	102	241
	Car	1184	657	1181	660	1841
Underlying illness and/or obesity	Unreported	498	349	596	251	847
	Reported	821	458	751	528	1279
Insurance	Insured	1319	0	990	329	1319
	Uninsured	0	807	357	450	807
Forgone care due to cost (past year)	No reported Forgone medical care	990	357	1347	0	1347
	Forgone medical care	329	450	0	779	779

Sample size for ages 20–64 includes n = 2126, with n = 1933 among those ages 20–64 after accounting for missing values of income. N = 2120 among those ages 20–64 after accounting for missing obs for English language proficiency. N = 2082 among those ages 20–64 after accounting for missing obs for Car Ownership. Note: Among those reporting incomes ≥ USD60 K, n = 0 reported *not* having a car. As such, car ownership is excluded from multivariable analyses, given models with and without income are compared. Additional Sub-analysis: Distribution of Ethnicity by Potential COVID-19 Severe Illness Risk included the following: n = 205/341 for non-Hispanic and n = 1074/1785 for Hispanic among those *with* Potential COVID-19 Severe Illness Risk.

**Table 2:** Unadjusted analyses for determinants of insurance status and forgone medical care due to cost.

		Uninsured			Forgone Medical Care Due to Cost		
		Ages 20–64 (n = 2126)			Ages 20–64 (n = 2126)		
		Odds Ratio	95% Confidence Interval		Odds Ratio	95% Confidence Interval	
Education	Less than College Degree versus College Degree	2.697 *	2.228	3.264	1.485 *	1.236	1.783
Ethnicity	Hispanic versus Not	2.723 *	2.059	3.599	1.299 *	1.015	1.664
Sex	Female versus Male	1.031	0.859	1.237	1.406 *	1.166	1.694
Age Group	20–44 versus 45–64	1.582 *	1.317	1.899	1.523 *	1.267	1.831
Underlying illness and/or obesity	Reported versus Unreported	0.796 *	0.666	0.951	1.669 *	1.388	2.008
Forgone care due to cost (past year)	Forgone Care versus not	3.793 *	3.147	4.572	n/a	n/a	n/a
Insurance	Uninsured versus insured	n/a	n/a	n/a	3.793 *	3.147	4.572
Excluded from multivariable models							
English language proficiency	Low versus High	4.356 *	3.284	5.777	0.874	0.663	1.153
Car Ownership	No Car versus Car	2.041 *	1.558	2.675	1.313	1.000	1.725
Income	<USD60 K versus ≥USD60 K	6.209 *	4.371	8.820	3.467 *	2.586	4.650

* significantly different using 95% Confidence Interval. Sample size for ages 20–64 includes n = 2126, with n = 1933 among those ages 20–64 after accounting for missing values of income. N = 2120 among those ages 20–64 after accounting for missing obs for English language proficiency. N = 2082 among those ages 20–64 after accounting for missing obs for Car Ownership. Note: Among those reporting incomes ≥ USD60 K, n = 0 reported *not* having a car. As such, car ownership is excluded from multivariable analyses, given models with and without income are compared.

**Table 3:** Adjusted analyses for determinants of insurance status and forgone medical care due to cost among working-age adults (age 20–64).

		Uninsured						Forgone Medical Care Due to Cost					
		Ages 20–64 (n = 2126)			Ages 20–64 (n = 1933)			Ages 20–64 (n = 2126)			Ages 20–64 (n = 1933)		
		Odds Ratio	95% Confidence Interval		Odds Ratio	95% Confidence Interval		Odds Ratio	95% Confidence Interval		Odds Ratio	95% Confidence Interval Odds Ratio	
Education	Less than College Degree versus College Degree	2.641 *	2.151	3.244	2.144 *	1.714	2.680	1.063	0.870	1.300	0.926	0.746	1.149
Ethnicity	Hispanic versus Not	2.493 *	1.847	3.364	2.316 *	1.688	3.179	0.895	0.685	1.169	0.875	0.662	1.156
Sex	Female versus Male	0.898	0.734	1.099	0.827	0.666	1.028	1.434 *	1.174	1.751	1.488 *	1.205	1.838
Age Group	20–44 versus 45–64	1.325 *	1.083	1.620	1.311 *	1.056	1.628	1.490 *	1.222	1.816	1.514 *	1.227	1.867
Income	<USD60 K versus ≥USD60 K	n/a	n/a	n/a	4.057 *	2.787	5.904	n/a	n/a	n/a	2.183 *	1.590	2.999
Underlying illness and/or obesity	Reported versus Unreported	0.604 *	0.493	0.739	0.587 *	0.472	0.729	2.007 *	1.642	2.454	1.926 *	1.557	2.382
Forgone care due to cost (past year)	Forgone Care versus not	3.956 *	3.239	4.833	3.676 *	2.969	4.551	n/a	n/a	n/a	n/a	n/a	n/a
Insurance	Uninsured versus insured	n/a	n/a	n/a	n/a	n/a	n/a	3.960 *	3.242	4.837	3.657 *	2.954	4.526

* significantly different using 95% Confidence Interval. Sample size for ages 20–64 includes n = 2126, with n = 1933 among those ages 20–64 after accounting for missing values of income. Note: Among those reporting incomes ≥ USD60 K, n = 0 reported *not* having a car. As such, car ownership is excluded from multivariable analyses, given that models with and without income are compared.

## Data Availability

Data are protected by IRB protocols as per the Texas A&M Institutional Review Board (IRB) and are not publicly available.
